# Circulating PD-L1 levels change during bevacizumab-based treatment in recurrent glioma

**DOI:** 10.1007/s00262-021-02951-2

**Published:** 2021-05-06

**Authors:** Maximilian J. Mair, Ayseguel Ilhan-Mutlu, Sahra Pajenda, Barbara Kiesel, Adelheid Wöhrer, Georg Widhalm, Karin Dieckmann, Christine Marosi, Ludwig Wagner, Matthias Preusser, Anna S. Berghoff

**Affiliations:** 1 Department of Medicine I, Division of Oncology, Medical University of Vienna, Vienna, Austria; 2 Department of Medicine III, Medical University of Vienna, Vienna, Austria; 3 Department of Neurosurgery, Medical University of Vienna, Vienna, Austria; 4 Department of Neurology, Division of Neuropathology and Neurochemistry, Medical University of Vienna, Vienna, Austria; 5 Department of Radiation Oncology, Medical University of Vienna, Vienna, Austria; 6 Christian Doppler Laboratory for Personalized Immunotherapy, Medical University of Vienna, Vienna, Austria

**Keywords:** Glioma, Soluble PD-L1, Liquid biomarker, Longitudinal measurement, Systemic inflammation

## Abstract

**Purpose:**

In primary brain tumors, the efficacy of immune-modulating therapies is still under investigation as inflammatory responses are restricted by tight immunoregulatory mechanisms in the central nervous system. Here, we measured soluble PD-L1 (sPD-L1) in the plasma of patients with recurrent glioblastoma (GBM) and recurrent WHO grade II–III glioma treated with bevacizumab-based salvage therapy.

**Methods:**

Thirty patients with recurrent GBM and 10 patients with recurrent WHO grade II–III glioma were treated with bevacizumab-based salvage therapy at the Medical University of Vienna. Prior to each treatment cycle, EDTA plasma was drawn and sPD-L1 was measured applying a sandwich ELISA with a lower detection limit of 0.050 ng/ml. Leukocyte counts and C-reactive protein (CRP) levels were measured according to institutional practice.

**Results:**

Median number of sPD-L1 measurements was 6 per patient (range: 2–24). At baseline, no significant difference in sPD-L1 concentrations was observed between WHO grade II–III glioma and GBM. Intra-patient variability of sPD-L1 concentrations was significantly higher in WHO grade II–III glioma than in GBM (*p* = 0.014) and tendentially higher in IDH-mutant than in IDH-wildtype glioma (*p* = 0.149) In WHO grade II–III glioma, sPD-L1 levels were significantly lower after one administration of bevacizumab than at baseline (median: 0.039 ng/ml vs. 0.4855 ng/ml, *p* = 0.036). In contrast, no significant change could be observed in patients with GBM.

**Conclusions:**

Changes in systemic inflammation markers including sPD-L1 are observable in patients with recurrent glioma under bevacizumab-based treatment and differ between WHO grade II–III glioma and GBM.

**Supplementary Information:**

The online version contains supplementary material available at 10.1007/s00262-021-02951-2.

## Introduction

Despite optimal surgical and adjuvant treatment, diffuse gliomas have a recurrence rate of up to 90% due to their infiltrative growth pattern [2]. However, the range of available treatment options at recurrence is very limited, underlining the urgent need for new therapeutic approaches. While immune-modulating therapies have revolutionized oncology, a clinically relevant efficacy in primary brain tumors such as glioma has not been observed so far. The CheckMate-143 trial comparing nivolumab with bevacizumab in recurrent glioma was unable to show a superiority of immune checkpoint inhibition [3]. However, a small subset of patients (objective response rate 7.8%) showed durable responses, underscoring the need for a more profound understanding of inflammatory subgroups in glioma potentially profiting from immune-modulating therapies [4].

Soluble programmed death receptor ligand 1 (sPD-L1) has been shown to correlate with prognosis and the response toward immune-modulating agents in a wide array of solid tumors [5–9]. Most probably, sPD-L1 is generated by proteolytic cleavage of membrane-bound PD-L1 on both tumor and immune cells. Cleavage may be generated by metalloproteinases such as ADAM10 or ADAM17 [10] whose expression has been described to correlate with poor prognosis in glioma [11]. Previously, we showed that sPD-L1 levels and detectability differ in different brain tumor entities and observed a correlation with survival in lower-grade glioma (LGG) and glioblastoma (GBM) [12]. Specifically, patients with GBM had longer overall survival (OS) in the presence of sPD-1 as compared to their counterparts (median OS 20.9 vs. 8.4 months, *p* = 0.006). In contrast, patients with LGG presented with worse OS when sPD-L1 could be detected (median OS 38.9 vs. 89.6 months, *p* = 0.028). These results suggest that the immune phenotype of LGG and GBM might differ, probably due to the immunosuppressive role of the metabolite 2-hydroxyglutarate (2-HG) in isocitrate dehydrogenase (IDH)-mutated tumors [13, 14]. Bevacizumab treatment is frequently used as a salvage treatment in patients with symptomatic glioma progression in need for steroid treatment [15]. Importantly, vascular endothelial growth factor signaling was previously shown to impact the efficacy of immune-modulating therapies in extracranial malignancies [16, 17]. Therefore, we aimed in the present study to investigate the longitudinal sPD-L1 concentrations as a systemic inflammatory marker in patients with recurrent LGG and GBM treated with bevacizumab-based therapy.

## Materials and methods

### Patient cohort

Patients ≥ 18 years with a recurrence of a histologically confirmed WHO grade II–IV glioma (as determined at the time of first surgery) planned for bevacizumab-based salvage treatment with bevacizumab (either 400 mg or 10 mg/kg body weight) every 2 weeks were included in this study. Blood samples were drawn before starting systemic bevacizumab-based salvage treatment and prior to each treatment cycle. Other inflammatory markers such as leukocyte counts and C-reactive protein (CRP) levels were concurrently measured at the Department of Laboratory Medicine of the Medical University of Vienna according to institutional practice. All patients were followed up until death. Patient data were stored in a password-secured database (FileMaker Pro Advanced/Server® 17, FileMaker Inc., Santa Clara, CA, USA) and were handled anonymously. The study was conducted according to the Declaration of Helsinki and its amendments as well as to local and institutional guidelines. The study was approved by the ethics committee of the Medical University of Vienna (approval no. 1315/2015).

### Immunohistochemistry

Anti-IDH1 R132H antibody (clone H09, Dianova GmbH, Hamburg, Germany) was used on a Ventana Benchmark Ultra immunostaining platform for evaluation of IDH mutation status [18]. Samples with specific staining in the tumoral region were classified as IDH-mutant (IDH-mt), while negative samples were classified as IDH-wildtype (IDH-wt).

### sPD-L1 ELISA

sPD-L1 levels were measured using a sandwich enzyme-linked immunosorbent assay (ELISA) as described previously [12]. Based on the standard dilution curve, a lower limit of sPD-L1 detection of 0.05 ng/ml was measured.

### Statistical analysis

The coefficients of variation (CV) were calculated to describe the variation of sPD-L1 concentrations within patients over time. Continuous variables were compared between groups by the Mann–Whitney U and Wilcoxon signed-rank test for unpaired and paired values, respectively. Overall survival (OS) was defined as the time between blood draw for sPD-L1 measurement and all-cause death and was compared applying the log-rank test. Results were considered significant at a *p *value of ≤ 0.05. As the study was aimed at the generation of hypotheses, no adjustment for multiple testing was performed [19].

Statistical analysis was performed using R 3.6.1 (The R Foundation for Statistical Computing, Vienna, Austria) with RStudio 1.2.1335 (RStudio Inc., Boston, MA, USA) and the packages “haven” (version 2.1.1), “ggplot2” (version 3.2.0), “gridExtra” (version 2.3), “GGally” (version 1.4.0), “labelled” (version 2.2.1), “scales” (version 1.0.0), and “survival” (version 2.44–1.1).

## Results

### Patients’ characteristics

Thirty patients with recurrent GBM and 10 patients with recurrent WHO grade II–III glioma were included in this study. The baseline characteristics of our cohort are listed in Table 1. Of note, IDH mutational status was available in 35/40 (87.5%) patients with 29/35 (82.9%) patients with IDH-wt and 6/35 (17.1%) with IDH-mt glioma. Baseline characteristics according to the presence/absence of IDH mutations are shown in Supplementary Table 1.

**Table 1:** Baseline characteristics

	Glioblastoma (*n* = 30)	Lower-grade glioma (*n* = 10)
*Gender*		
Male	24 (80.0%)	8 (80.0%)
Female	6 (20.0%)	2 (20.0%)
*Age at first diagnosis (years)*		
Median (range)	52 (20–75)	47.5 (27–56)
*IDH mutation*		
IDH-wt	24/30 (73.3%)	5/10 (50.0%)
IDH-mt	1/30 (3.3%)	5/10 (50.0%)
Unknown	5/30 (23.3%)	0/10 (0.0%)
*MGMT promoter methylation status*		
Methylated	4 (13.3%)	–
Unmethylated	8 (26.7%)	–
Unknown	18 (60.0%)	10 (100.0%)
*Treatment*		
Bevacizumab alone	14 (46.7%)	5 (50.0%)
Bevacizumab + alkylating agent	9 (30.0%)	4 (40.0%)
Bevacizumab + tyrosine kinase inhibitor	7 (23.3%)	1 (10.0%)
*Bevacizumab dosage*		
400 mg absolute	16 (53.3%)	8 (80.0%)
→ Corresponding to mg bevacizumab per kg body weight; median, range	5.1 (4–8.2)	4.7 (4–6.7)
10 mg/kg body weight	14 (46.7%)	2 (20.0%)
*Documented dexamethasone use during study*		
Yes	18 (60.0%)	3 (30.0%)
No	12 (40.0%)	7 (70.0%)
*sPD-L1*		
Detection rates at baseline	19 (63.3%)	8 (80.0%)
Median (range) of positive samples at baseline [ng/ml]	0.321 (0.080–42.110)	0.658 (0.060–2.250)
Median number of measurements per patient	6 (2–16)	5 (2–24)
Median time to bevacizumab treatment in months (range)	12.1 (5.8–88.6)	37.5 (12.1–81.0)
Median overall survival from first diagnosis (months)	21.5 (95% CI: 17.9–31.9)	53.2 (95% CI: 32.2–72.4)
Median overall survival from first bevacizumab treatment (months)	5.3 (95% CI: 4.7–7.9)	6.8 (95% CI: 3.3–14.6)

### sPD-L1 concentrations

**Fig. 1:**
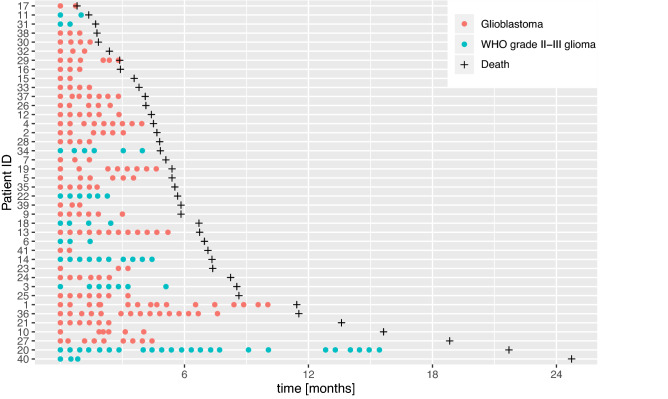
Available measurements over time (•) and overall survival from first bevacizumab treatment ( +) in the study cohort

In patients with GBM, the median number of sPD-L1 measurements per patient was 6 (range: 2–16), while in median 5 measurements (range: 2–24) were available in patients with WHO grade II–III glioma. Figure 1 shows a timeline which illustrates survival and the available measurements from the time of treatment initiation.

At baseline, sPD-L1 could be detected in 19/30 (63.3%) patients with GBM, whereas sPD-L1 could be found in the plasma of 8/10 (80.0%) patients with WHO grade II–III glioma (*p* = 0.451, Fisher’s exact test). The median sPD-L1 concentration in sPD-L1-positive samples at baseline was 0.321 ng/ml (range: 0.080–42.110 ng/ml) in patients with GBM and 0.658 ng/ml (range: 0.060–2.250 ng/ml) in patients with WHO grade II–III glioma (*p* = 0.465, Mann–Whitney U test; Supplementary Fig. 1A).

In 18/29 (62.1%) of IDH-wildtype (IDH-wt) patients, sPD-L1 could be detected at baseline while this was the case in 5/6 (83.3%) IDH-mutated (IDH-mt) patients (*p* = 0.64, Fisher’s exact test). Median sPD-L1 concentration in sPD-L1-positive patients at baseline was 0.2795 ng/ml (range: 0.057–3.383 ng/ml) in IDH-wt and 0.563 ng/ml (range: 0.127–2.245 ng/ml) in patients with IDH-mt tumors (*p* = 0.551, Mann–Whitney U test; Supplementary Fig. 1B).

### Longitudinal changes in sPD-L1 concentrations over time

**Fig. 2:**
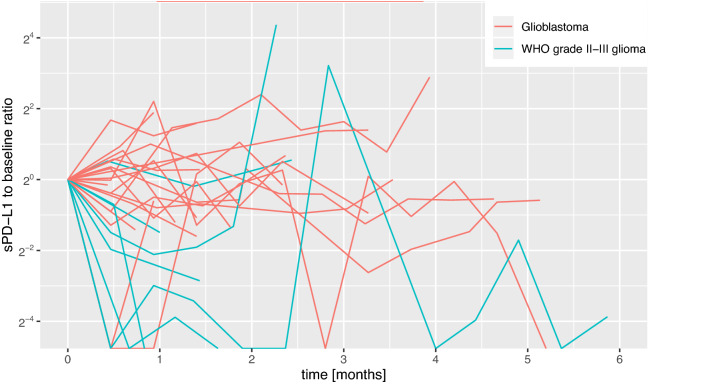
Spider plot showing the ratios between sPD-L1 concentrations at each timepoint and baseline

Median CV was 0.63 (range: 0–2.606) in GBM and 1.766 (range: 0.252–3.958) in WHO grade II–III glioma. Intra-patient variation of sPD-L1 concentrations was significantly higher in patients with WHO grade II–III glioma compared to patients with GBM (*p* = 0.014, Mann–Whitney U test; Fig. 2). Moreover, a trend toward higher sPD-L1 variation in IDH-mt (median: 1.71, range: 0.25–3.96) glioma was observed as compared to IDH-wt tumors (median: 0.77, range: 0–3.162; *p* = 0.149). No difference in intra-patient variation of sPD-L1 could be observed between patients who received dexamethasone during their treatment (median: 0.64, range: 0–2.64) and those who did not (median: 0.93, range: 0–3.96; *p* = 0.279). Furthermore, CV did not differ based on whether patients were treated with bevacizumab alone, bevacizumab + alkylating agent or bevacizumab + tyrosine kinase inhibitor (*p* = 0.764, Kruskal–Wallis test). A numerical trend towards higher intra-patient variation of sPD-L1 levels was observed in patients who received bevacizumab 10 mg/kg body weight as compared to patients where 400 mg bevacizumab were administered (*p* = 0.103, Mann–Whitney U test).

**Fig. 3:**
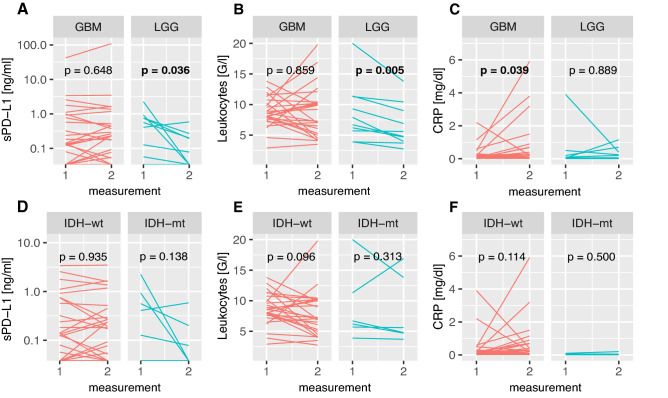
sPD-L1 concentrations (**a**/**d**), leukocyte counts (**b**/**e**) and CRP concentrations (**c**/**f**) at the first and second measurements in glioblastoma (GBM, **a**-**c**, left panel) and WHO grade II–III glioma (lower-grade glioma = LGG, **a-c**, right panel) as well as IDH-wildtype (IDH-wt, **d-f**, left panel) and IDH-mutated (IDH-mt, **d-f**, right panel) glioma. P values as determined by Wilcoxon signed-rank test

In GBM, there was no significant difference between pre-treatment levels of sPD-L1 and sPD-L1 concentrations before cycle 2 of bevacizumab-based treatment (median sPD-L1 pre-treatment = 0.13 ng/ml vs. before cycle 2 = 0.2025 ng/ml, *p* = 0.648, Wilcoxon signed-rank test; Fig. 3a, left panel). In contrast, sPD-L1 levels significantly decreased in WHO grade II–III glioma (median sPD-L1 pre-treatment = 0.4855 vs. before cycle 2 = 0.039; *p* = 0.036; Fig. 3a, right panel). Similarly, leukocyte counts significantly diminished in WHO grade II–III glioma (median leukocyte counts pre-treatment = 7.3 G/l vs. before cycle 2 = 5.2 G/l, *p* = 0.005; Fig. 3b, right panel), while there was no significant change in GBM (median pre-treatment = 8.6 G/l vs. before cycle 2 = 7.2 G/l, *p* = 0.859; Fig. 3b, left panel). In contrast, CRP levels increased in patients with GBM (median pre-treatment = 0.1 mg/dl vs. before cycle 2 = 0.195 mg/dl, *p* = 0.039; Fig. 3c, left panel), while no significant change could be observed in WHO grade II–III glioma (median pre-treatment = 0.06 mg/dl vs. before cycle 2 = 0.13 mg/dl, *p* = 0.886; Fig. 3c, right panel).

No significant differences could be detected between pre-treatment and after cycle 1 in both IDH-wt and IDH-mt tumors in terms of sPD-L1, leukocyte counts and CRP levels. However, a numerical trend toward a decrease of sPD-L1 in IDH-mt glioma (median pre-treatment = 0.4855 ng/ml vs. before cycle 2 = 0.039 ng/ml, *p* = 0.138; Fig. 3d, right panel) was observed. Furthermore, in IDH-wt tumors, leukocyte counts tendentially decreased (median pre-treatment = 8.6 G/l vs. before cycle 2 = 7.03 mg/dl, *p* = 0.096; Fig. 3e, left panel) and CRP levels tendentially increased (median pre-treatment = 0.11 mg/dl vs. before cycle 2 = 0.26 mg/dl, *p* = 0.114; Fig. 3f, left panel).

### Survival analysis—prognostic impact of sPD-L1

With respect to overall survival (OS), there were no significant differences between sPD-L1 positive and negative patients at baseline in the GBM (*p* = 0.56, log-rank test) and WHO grade II–III glioma subgroups (*p* = 0.68) as well as in confirmed IDH-wt cases (*p* = 0.63, Supplementary Fig. 2A-C).

Furthermore, the prognosis of patients with decreasing sPD-L1 in the course of bevacizumab-based therapy did not differ from those where sPD-L1 increased between baseline and the second measurement in both the GBM (*p* = 0.77) and WHO grade II–III sub-cohorts (*p* = 0.9) (Supplementary Fig. 2D/E). Similarly, there was no difference in OS in IDH-wt glioma according to the change in sPD-L1 levels after the first treatment cycle (*p* = 0.65) (Supplementary Fig. 2F). Survival analysis in the IDH-mt subgroup was not performed due to small sample sizes.

## Discussion

In the present study, we analyzed the time course of sPD-L1 as a surrogate marker for systemic inflammation in patients with recurrent glioma receiving bevacizumab-based treatment. As shown previously [12], sPD-L1 is a detectable marker in brain tumors and correlates with local and systemic inflammatory parameters. Here, we found a significant decrease in sPD-L1 levels upon initiation of bevacizumab-based salvage treatment in WHO grade II–III glioma. This change was accompanied by alterations of other established markers of systemic inflammation such as leukocyte counts and CRP levels.

Timely changes of sPD-L1 and other inflammatory parameters in bevacizumab-treated patients with glioma suggest that tumor–immune system interactions are observable on a systemic level. Although gliomas rarely show extracranial metastases and present with a growth pattern restricted to the CNS, several studies previously suggested that systemic inflammation is altered by glioma. Erythrocyte sedimentation rate, CRP levels and the neutrophil-to-lymphocyte ratio were shown to correlate with survival prognosis [20, 21]. Further, an association of systemic inflammation markers such as leukocyte, neutrophil and platelet counts as well as CRP levels with markers of the local tumor microenvironment and overall survival was reported [22]. Indeed, the variability in the systemic inflammation could be an explanation for the so far lacking clinical efficacy of immuno-modulatory therapies in glioma. Systemic inflammation as measured by CRP, neutrophil-to-lymphocyte ratio or recently also sPD-L1 was shown to correlate with the likelihood of response to immune checkpoint inhibitors in extracranial malignancies [23]. Profiling of systemic tumor–immune system interactions could therefore allow for a rational selection of patients who potentially benefit from immunotherapy. Notably, although the CheckMate-143 trial failed to meet its primary endpoint, few durable responses were observed in the nivolumab arm, suggesting that a small subgroup of patients with GBM benefits from immunotherapy [3].

There was higher inter-patient variability of sPD-L1 concentrations in WHO grade II–III glioma than in GBM as determined by the CV. Although not statistically significant, we could detect tendentially higher intra-patient variability in IDH-mt glioma than in IDH-wt tumors. WHO grade II–III gliomas more frequently display IDH mutations leading to an abundant production of the oncometabolite 2-HG exerting local immune-modulating effects. Here, we provide further data on distinct glioma–immune system interactions on systemic level, adding further to the evidence that tumor–immune system interactions differ between glioma subtypes.

In the present study, only patients receiving bevacizumab-based treatment were included. It is well known that therapies targeting the vascular endothelial growth factor (VEGF) axis interact with local and systemic inflammation. Specifically, VEGF inhibition regulates the infiltration of immune cells to the tumor microenvironment by normalizing the tumor vasculature and altering the endothelial expression of cell adhesion molecules [24]. Furthermore, VEGF along with other immunosuppressive factors exerts systemic immunosuppressive effects hampering effective antitumor immune responses [25]. Recently, the results of a phase II trial of pembrolizumab with bevacizumab in recurrent glioma were presented [26]. However, combination therapy was not superior in terms of efficacy as compared to historical bevacizumab monotherapy controls. With regard to our data, it remains elusive whether the observed effects on systemic inflammation are bevacizumab-related or rather due to glioma progression, as bevacizumab exerts only minor antitumoral efficacy in glioma and is mainly used for symptomatic control without significant benefit in terms of overall survival [27].

Clearly, our study has several limitations which are mainly due to its exploratory, retrospective design and the small patient number resulting in limited statistical power to detect significant alterations especially in subgroup analyses. The variability in number of measurements per patient with as few as two measurements available in some patients impeded statistical testing for time-dependent effects. Moreover, although we applied the same sandwich ELISA as previously published [12], the method has not been systemically validated with other assays. Lastly, with only 6 IDH-mutant glioma cases, further analyses with respect to molecular subtypes could not be performed.

In conclusion, our data show that tumor–immune system interactions are observable on a systemic level in patients with recurrent glioma. sPD-L1 concentrations change over time in a cohort treated with bevacizumab-based salvage treatment and correlations with other markers of systemic inflammation could be detected, although the mechanistic foundations remain to be elucidated. Our data underscore the need for comprehensive panels of immune-related biomarkers for the conception of prospective immunotherapy trials in CNS tumors. Furthermore, our data add to the available evidence that tumor–immune system interactions differ between glioma subtypes which should be considered in future studies on immune-modulating agents and combinations thereof in glioma.

### Supplementary Information

Below is the link to the electronic supplementary material.

Supplementary file1 (PDF 1005 KB)

## Abbreviations

2-HG: 2-Hydroxyglutarate
CV: Coefficient of variation
CNS: Central nervous system
CRP: C-reactive protein
ELISA: Enzyme-linked immunosorbent assay
GBM: Glioblastoma multiforme
IDH: Isocitrate dehydrogenase
LGG: Lower-grade glioma (WHO grade II–III glioma)
OS: Overall survival
PD-L1: Programmed death receptor ligand 1
sPD-L1: Soluble PD-L1
VEGF: Vascular endothelial growth factor
WHO: World Health Organization
