# Supplementary material for: Circulating PD-L1 levels change during bevacizumab-based treatment in recurrent glioma
Source: Cancer Immunol Immunother. 2021 May 6;70(12):3643–50. doi: 10.1007/s00262-021-02951-2 (PMC8571215; doi:10.1007/s00262-021-02951-2)
Supplement: Supplementary file 1 — Supplementary file1 (PDF 1005 KB) [file 262_2021_2951_MOESM1_ESM.pdf]

	IDH-wildtype (n = 29)	IDH-mutated (n = 6)
**Gender**		
**- Male**	22 (75.9%)	5 (83.3%)
**- Female**	7 (24.1%)	1 (16.7%)
**Age at first diagnosis (years)**		
**- Median (range)**	51 (20 - 75)	30.5 (24 - 56)
**WHO Grades**		
**- Grade II - III**	5 (17.2%)	5 (83.3%)
**- Grade IV**	24 (82.8%)	1 (16.7%)
**MGMT promoter methylation status**		
**- methylated**	4 (13.8%)	-
**- unmethylated**	8 (27.6%)	-
**- unknown**	17 (58.6%)	6 (100.0%)
**Treatment**		
**- bevacizumab alone**	16 (55.2%)	2 (33.3%)
**- bevacizumab + alkylating agent**	6 (20.7%)	3 (50.0%)
**- bevacizumab + tyrosine kinase inhibitor**	7 (24.1%)	1 (16.7%)
**Bevacizumab dosage**		
**- 400 mg absolute**	16 (55.2%)	6 (80.0%)
→ corresponding to mg bevacizumab per kg body weight; median, range	5.1 (4 - 8.2)	5.6 (4 - 6.7)
**- 10 mg/kg body weight**	13 (44.8%)	0 (20.0%)
**Documented dexamethasone use during study**		
**- yes**	15 (51.7%)	2 (33.3%)

**- no**	14 (48.3%)	4 (66.7%)
**sPD-L1**		
**- Detection rates at baseline**	18 (62.1%)	5 (83.3%)
**- Median (range) of positive samples at baseline [ng/ml]**	0.2795 (0.057 – 3.383)	0.563 (0.127 – 2.245)
**- Median number of measurements per patient**	6 (2 - 19)	8 (5 - 24)
**Median time to bevacizumab treatment in months (range)**	15.3 (5.9 – 88.6)	37.5 (5.8 – 76.1)
**Median overall survival from first diagnosis (months)**	21.6 (95% CI: 18.7 – 25.1)	53.2 (95% CI: 35.1 – n.r.)
**Median overall survival from first bevacizumab treatment (months)**	5.4 (95% CI: 4.5 – 7.2)	18.8 (95% CI: 6.7 – n.r.)

Supplementary Table 1. Baseline characteristics according to IDH mutational status available in 31/40 (77.5%) patients

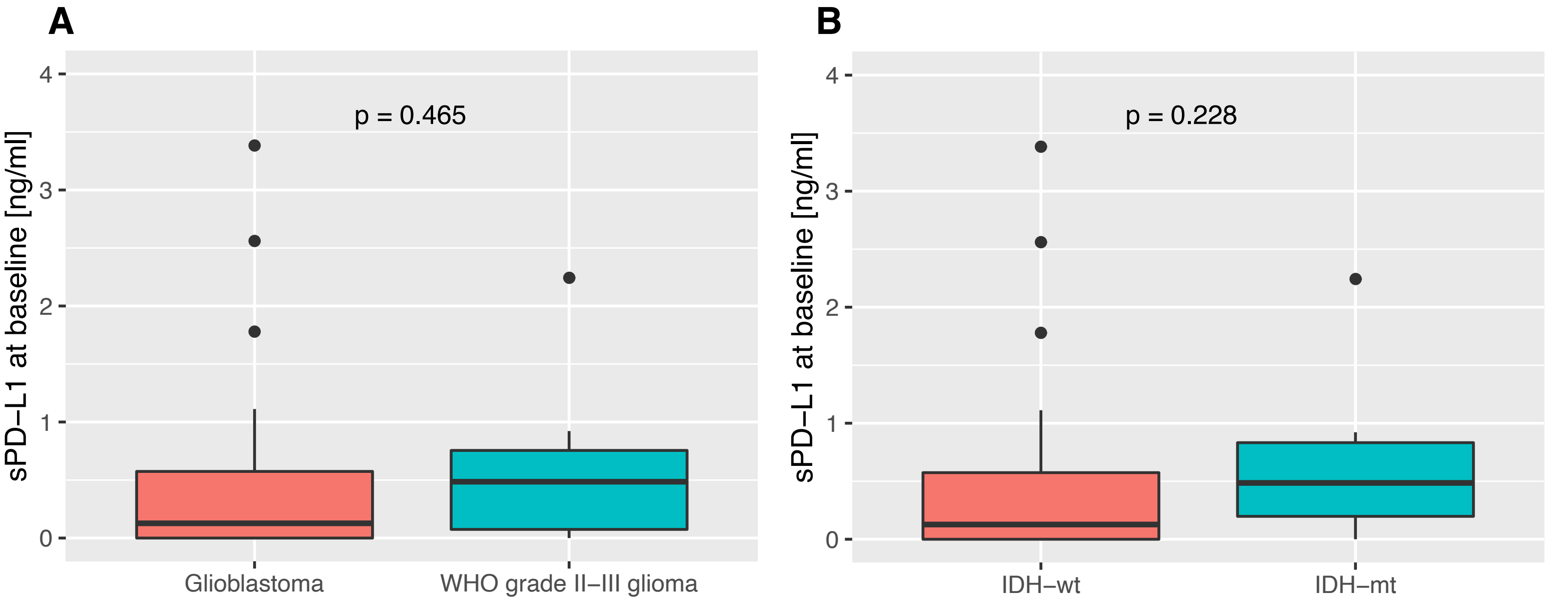

Supplementary Fig. 1. sPD-L1 levels at baseline in (A) glioblastoma and WHO grade II-III glioma and (B) IDH-wildtype (IDH-wt) and IDH-mutant (IDH-mt) tumors.

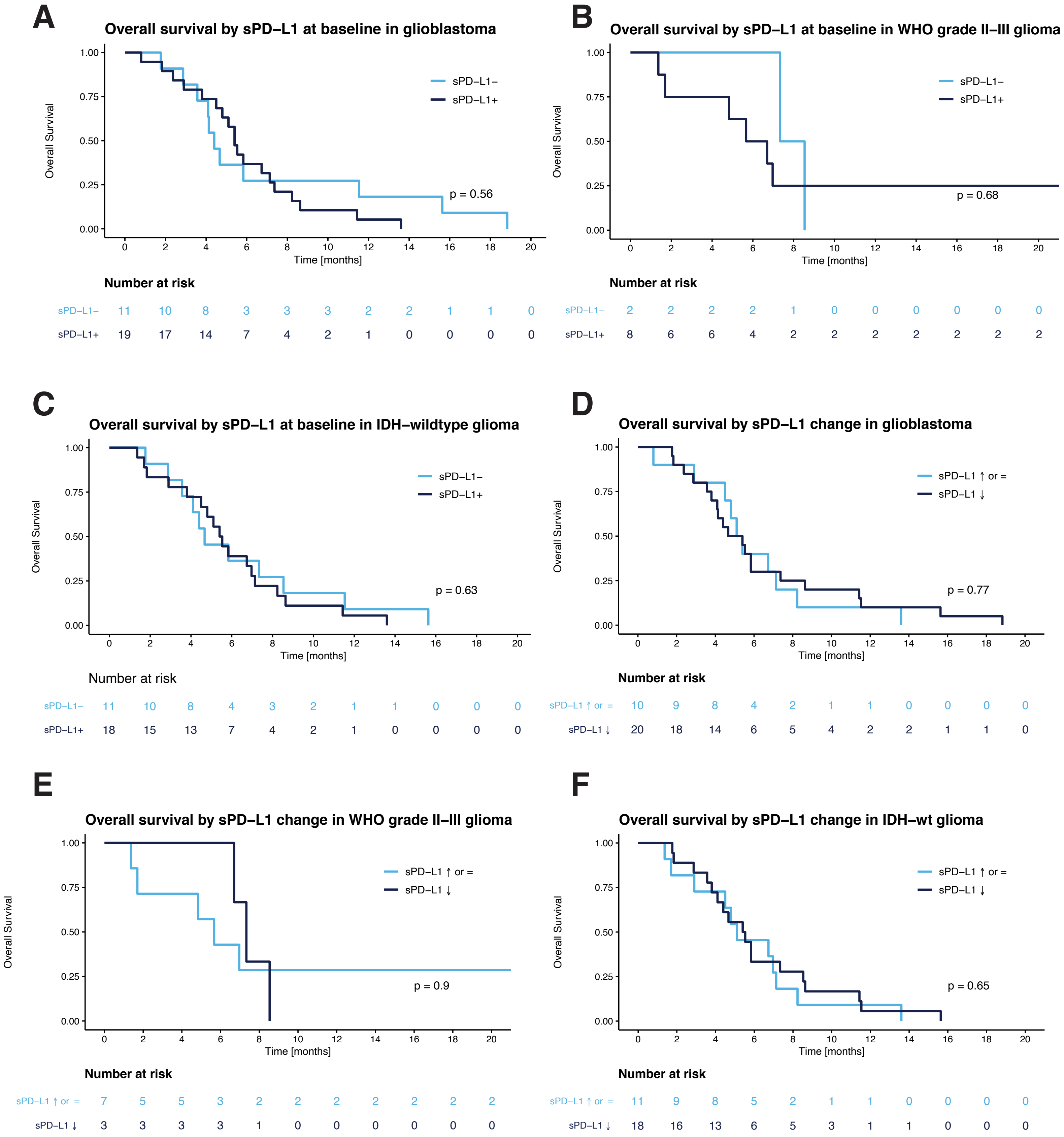

Supplementary Fig. 2. Overall survival according to sPD-L1 detectability at baseline in (A) glioblastoma, (B) WHO grade II-III glioma, (C) IDH-wildtype glioma; Overall survival according to sPD-L1 change in (D) glioblastoma, (E) WHO grade II-III glioma, (F) IDH-wildtype glioma.
